# Comparative Immunogenicity in Rabbits of the Polypeptides Encoded by the 5′ Terminus of Hepatitis C Virus RNA

**DOI:** 10.1155/2015/762426

**Published:** 2015-11-02

**Authors:** Irina Sominskaya, Juris Jansons, Anastasija Dovbenko, Natalia Petrakova, Ilva Lieknina, Marija Mihailova, Oleg Latyshev, Olesja Eliseeva, Irina Stahovska, Inara Akopjana, Ivars Petrovskis, Maria Isaguliants

**Affiliations:** ^1^Latvian Biomedical Research and Study Center, Ratsupites Street 1, Riga LV-1067, Latvia; ^2^N. F. Gamaleja Research Center of Epidemiology and Microbiology, Gamaleja Street 18, Moscow 123098, Russia; ^3^Department of Microbiology, Tumor and Cell Biology, Karolinska Institutet, Nobels Väg 16, 17177 Stockholm, Sweden; ^4^Riga Stradins University, Dzirciema Street 16, Riga LV-1007, Latvia

## Abstract

Recent studies on the primate protection from HCV infection stressed the importance of immune response against structural viral proteins. Strong immune response against nucleocapsid (core) protein was difficult to achieve, requesting further experimentation in large animals. Here, we analyzed the immunogenicity of core aa 1–173, 1–152, and 147–191 and of its main alternative reading frame product F-protein in rabbits. Core aa 147–191 was synthesized; other polypeptides were obtained by expression in *E. coli*. Rabbits were immunized by polypeptide primes followed by multiple boosts and screened for specific anti-protein and anti-peptide antibodies. Antibody titers to core aa 147–191 reached 10^5^; core aa 1–152, 5 × 10^5^; core aa 1–173 and F-protein, 10^6^. Strong immunogenicity of the last two proteins indicated that they may compete for the induction of immune response. The C-terminally truncated core was also weakly immunogenic on the T-cell level. To enhance core-specific cellular response, we immunized rabbits with the core aa 1–152 gene forbidding F-protein formation. Repeated DNA immunization induced a weak antibody and sustained proliferative response of broad specificity confirming a gain of cellular immunogenicity. Epitopes recognized in rabbits overlapped those in HCV infection. Our data promotes the use of rabbits for the immunogenicity tests of prototype HCV vaccines.

## 1. Introduction

Nucleocapsid (core) protein of hepatitis C virus (HCV) is the most conserved HCV antigen capable of inducing strong broadly cross-reactive responses, and therefore an attractive component of a genotype-non-restricted HCV vaccine. As such, it has been included in a number of HCV vaccine candidates including ones reaching primate trials [1]. The responses observed were described as limited. In immunizations, HCV core demonstrated features of a weak immunogen capable of inducing mainly CTL and low or no CD4+ T-cell responses with moderate IFN-gamma, weak IL-2 production, and no antibodies [2, 3]. In primate trials, HCV core induced stable low-level T-cell response of CD4+ and CD8+ T-cells manifested by IFN-gamma, but no IL-2 or IL-4 responses, weak T-cell proliferation, and low titer of core-specific antibodies [4–8]. Attempts to achieve a more efficient anticore immune response met with difficulties [9–11] even when using viral vectors [12].

Interestingly, in natural infection HCV core acts as a strong humoral immunogen inducing an early potent antibody production, but limited cellular response. Furthermore, in patients developing chronic infection, antibody response to HCV core protein continues to expand, whereas the cellular responses shrink [13]. This scenario points at a limited (low to no) protective potential of core-specific humoral responses. At the same time, in primate trials, the responses to structural HCV proteins including core were shown to significantly correlate with primate protection against HCV challenge (whereas no protection was rendered by immunizations with nonstructural proteins) [1]. This indicates a potential positive input of anticore response (moderate as it was) on the observed protection effects, emphasizing the necessity to achieve an effective core-specific cellular response. Achieving stronger core-specific responses required the addition of recombinant HCV core protein or core-derived peptides [9, 14], involvement of the Th2-tilting carriers as HBcAg [15], or coadministration of cytokines such as IL-2, IL-4, or granulocyte-macrophage CSF [16], altogether pointing at the necessity of a shift towards the Th2-type T-helper cell response. Interestingly, these particular responses (of CD4+ T-cells) are involved in the spontaneous clearance of HCV infection, contrary to the CTL response reported to be stunned and ineffective [13, 17].

The reasons for a deficiency of such response in natural infection are not yet fully understood. Several explanations can be named, firstly, the well-known immunomodulating properties of HCV core protein [18–20]. The other reason could be the abundance of HCV core as an antigen. The core antigen quantity correlates with the virus load and can reach high levels in chronic HCV infection [21, 22], whereas the induction of potent cellular response appears to rely on the low immunogen doses [23]. An interference was also implied by the proteins translated from the HCV alternative reading frames (ARFs) [24, 25]. Most of the core gene products appear to be contaminated with the proteins translated from the HCV alternative reading frames (ARFPs) [24, 25]. The difference in anti-F response between chronic and self-limiting infection, the cross-reactivity irrespective of genotype, and the correlation of anti-F response to the response against other structural and nonstructural HCV antigens pointed at the immune response to F-protein as an integral part of the natural HCV infection [26]. As in case of HCV core, strong antibody response to F-protein correlates with the chronical course of HCV infection [27]. Kong et al. showed recently that presence of anti-F-specific antibodies negatively correlates with HCV RNA viral load suggesting that F-protein may participate in viral clearance [28]. However, other results suggest the potential involvement of F-protein (as of core antigen) in increasing the frequency of CD4+CD25+FoxP3+ T-cell-like population and IL-10-producing CD4+CD25+ T-cells [24] and biased cytokine responses (significantly decreased IFN-*γ* and/or IL-2 and significantly increased IL-4 and/or IL-5 levels) [25] predisposing to persistent HCV infection. ARFPs may induce some of the negative effects ascribed to HCV core [29] and also sidetrack the immune response away from HCV core. The true role of anti-ARFP responses in resistance to viral infection or vaccine protection is yet unknown.

In this work we aimed to directly compare immunogenicity of protein products encoded by 5′ end of HCV RNA in comparatively large animals, namely, in rabbits, which have numerous advantages over mice and are regularly used prior to testing vaccines in primates. Specifically, we compared immunogenicity of the main form of HCV core, core aa 1–173, its shorter form core aa 1–152, the C-terminal core aa 147–191, and F-protein as an ARFP form with the longest unique protein domain. All polypeptides generated extremely potent humoral response resembling that in chronic HCV infection. At the same time, a synthetic gene for the C-terminally truncated HCV core forbidding F-protein synthesis generated a sustained T-cell and only low antibody response indicating a clear shift towards cellular immunity deemed essential for an effective HCV vaccine.

## 2. Materials and Methods

### 2.1. *E. coli* Strains

*E. coli* strain DH5*α* [F^−^ *gyrA96* (Nal^r^)* recA1 relA1 endA1 thi-1 hsdR17* (r_k_^−^m_k_^+^)* glnV44 deoR* Δ*(laczya-argF) U169* [Φ80dΔ*(lacZ)M15*] was used for genetic manipulations and* E. coli* strains JM109 [F′* traD36 proA* ^*+*^ *B* ^*+*^ *lacI* ^*q*^ *Δ(lacZ)M15/Δ(lac-proAB) glnV44* e14^−^ (McrA^−^)* gyrA96* (Nal^r^)* recA1 relA1 endA1 thi-1 hsdR17* (r_k_^−^m_k_^+^)] and BL21(DE3) [F^−^ *ompT dcm lon hsdS* (r_B_^−^m_B_^−^)* gal λ(DE3)*] were used for expression.

### 2.2. Plasmids for Expression of HCV Core

Fragment corresponding to HCV core 1–173 aa was obtained by polymerase chain reaction (PCR) using cDNA of HCV AD78 isolate genotype 1b (GenBank accession number AJ132996 [30]) as a template and two primers: forward 5′-GATCCATGGGCACGAATCCTAAACCTCA contained NcoI site and reverse 5′-GTGATGAGATCTAGAGCAACCGGGCAGATTCCCTGTTGCA contained BglII site. Second codon AGC from AJ132996 was substituted for GGC and thus gave us S to G substitution. NcoI/BglII PCR fragment was ligated into NcoI/BglII pQE-60 plasmid (Qiagen). The resulting plasmid was named pQE/core 173 (GenBank accession number KT824963).

Amplification of the DNA fragment corresponding to 1–10 aa of core and in +1 frame of the core from aa 11 to aa 143 and two additional aa (LE) was performed by PCR using 5′-GAGCATATGAGCACGAATCCTAAACCTCAAAGAAAACCAAACGTA as forward primer and 5′-GTGGTGCTCGAGTGGTGGCGCCGACGAGCGGA as reverse primer; harboring NdeI and XhoI restriction sites, respectively, was done from plasmid bearing HCV core fragment corresponding to 1–191 aa of HCV 1b isolate 274933RU (GenBank accession #AF176573 [31]). After amplification and treatment with restriction endonucleases NdeI and XhoI fragment was ligated into NdeI/XhoI pET22b(+). pET22b(+) plasmid contains T7 promoter and 6xHis-tag coding sequence at 3′ end of the cloned DNA fragment. The resulting plasmid was named pET22/ARFP.

### 2.3. Sources of HCV-Derived Peptides

Polypeptide representing aa 147–191 of HCV core VARALAHGVRVLEDGVNYATGNLPGCSFSIFLLALLSCLTIPASA (core 147–191) was purchased from GL Biochem (Shanghai, China) and was at least 70% pure by HPLC.

HCV core-derived synthetic peptides used in analysis of immune response were purchased from GL Biochem (Shanghai, China) or kindly provided by Mati Sällberg (Karolinska Institutet, Sweden); and F-protein-derived peptides were purchased from Peptron (South Korea). Peptides were purified by HPLC to 70% purity. The list of synthetic peptides used is given in Table 1.

### 2.4. Sources of HCV Polyproteins

Expression of HCV core aa 1–152 (core 1–152) and core aa 1–173 (core 1–173) was carried out in the* E. coli* strain JM109 as was described earlier in [32] and [33], respectively.

F-protein was expressed in* E. coli* BL21(DE3) transformed with pET22/ARFP. Transformed bacterial cells were grown at 37°C in 2x TY medium (16 g/L bacto-peptone (Difco), 10 g/L yeast extract (Difco), and 5 g/L NaCl), supplemented with 100 *μ*g/mL ampicillin, to an OD 540 of 0.8–1.0, and protein expression was induced with 0.2 mM IPTG. Induction was continued for 4 h at 37°C; after that cells were sedimented by low-speed centrifugation (10 min at 4,000 ×g) and frozen at −20°C. Frozen biomass was thawed and suspended in 10 volumes of 8 M urea containing 100 mM Tris-HCl, pH 8.0, and ultrasonicated with ten 60 s ultrasound pulses of 22 kHz. After ultrasonication incubation on ice was continued for 60 min. After clarification (30 min at 10000 ×g), supernatant was collected and dithiothreitol (DTT) was added to 100 mM and incubation was continued overnight by shaking on rotary shaker at 4°C. After repeated clarification (30 min at 10000 ×g) before loading onto immobilized-metal affinity chromatography (IMAC) Ni-superflow agarose (Qiagen, Hilde, Germany), buffer exchange was performed with Sephadex G-25 column to replace 100 mM DTT with 5 mM *β*-mercaptoethanol (*β*-ME). The recombinant protein was purified by IMAC under denaturing conditions (8 M urea, 5 mM *β*-ME, 100 mM Tris-HCl, and pH 8.0) according to the manufacturer's instructions. F-protein containing fractions were pooled, and purified protein was diluted to final concentration of 0.5 mg/mL. The proteins were subsequently dialyzed two times (overnight and for 4 to 6 h) using refolding buffer I (2 M urea, 100 mM PB (Na_2_HPO_4_: 94.7 mM; NaH_2_PO_4_: 5.3 mM), pH 8.0, 0.5 M arginine, 5 mM glutathione reduced [GSH], and 0.5 mM glutathione oxidized [GSSG]) and refolding buffer II (100 mM PB, pH 8.0, 0.5 M arginine, 5 mM GSH, and 0.5 mM GSSG) and then PBS with 10% glycerin. Soluble proteins were concentrated using Amicon Ultra-15 10 K centrifugal filter device 10,000 MWCO (Millipore, Ireland). Its purity according to Coomassie blue staining of the SDS-PAGE gel was 95%.

### 2.5. SDS-PAGE and Western Blot Analysis

The purified proteins were analyzed on 15% SDS-PAGE by standard procedures (under denaturing conditions). Proteins were transferred to nitrocellulose membrane (Thermo Scientific). After blocking, the membranes were probed with rabbit antibodies specific to HCV core [34] or anti-core 1–173 or F-protein antibodies obtained here (see Section 2.6) diluted 1 : 10000, followed by a protein A horseradish peroxidase-conjugated antibody diluted 1 : 1000. Detection was performed with the DAB Substrate Kit (Thermo Scientific) according to the manufacturer's protocol.

### 2.6. Immunization of Rabbits

All animal experiments were performed in accordance with the Russian Federation law and were approved by the institutional ethical committee for animal experiments. Moscow strain of Chinchilla grey rabbits (female, 2-month-old, 1.5 to 1.8 kg) was obtained from the laboratory animal breeders “Manikhino” (settlement Manikhino, Ivanovskoe, Moscow region, Russia) or “Krolinfo” (Orekhovo-Zuevo, Moscow region, Russia, http://krolinfo.umi.ru). The animals were maintained at 20 to 22°C and a relative humidity of 50% ± 10% on a 12 h light/dark cycle, fed with commercial rodent chow and herbal vitamin flour (“Krosha” and “Meadow grass,” both from Zoomir, Russia), and provided with tap water* ad libitum*. The treatment of animals was in accordance with regulations outlined in the USDA Animal Welfare Act and the conditions specified in the guide for care and use of laboratory animals [35].

In protein immunizations, groups of two Chinchilla rabbits were immunized with injections of recombinant core 147–191 (numbers 87, 88), core 1–152 (89/4, 90/5), F-protein (91, 92), and core 1–173 (93, 94) or mock-immunized with PBS (95, 96). At week 0 animals were administered 100 *μ*g of the respective polypeptides in 400 *μ*L PBS mixed (1 : 1 v/v) with the complete Freund Adjuvant (CFA) and a week later (week 1) with 100 *μ*g of the respective polypeptides in 400 *μ*L PBS mixed (1 : 1 v/v) with the incomplete Freund Adjuvant (IFA). Injections were done subcutaneously at four sites along the back. Animals were boosted three times with one-month intervals by the intravenous injections of 50 *μ*g of polypeptides in 200 *μ*L PBS mixed with IFA (1 : 1 v/v). Control animals (95, 96) received the adjuvants mixed with PBS. Rabbits were bled from the ear vein two weeks after each immunization. Sera were prepared and stored at −20°C until further analysis. A portion of blood was collected in the heparinized Vacutainer tubes, and peripheral mononuclear cells (PBMCs) were isolated by Ficoll Paque gradient centrifugation.

DNA immunizations were performed with pUC8-based plasmid encoding core aa 1–152 [36] under the control of CMV promoter and HPV16 polyA [37] (DNAcore152). For this, four rabbits (nn 98, 99, 101, and 102) were injected with 90 *μ*g DNAcore152 in 400 *μ*L water intramuscularly in tibialis anterior on weeks 1 and 2. Two rabbits (101, 102) were further boosted with 90 *μ*g DNAcore152 in 400 *μ*L on weeks 5 (boost 1), 18 (2), 37 (3), and 54 (4). Control rabbits (43, 44) were immunized with empty pCMV vector [37] administered repeatedly along the same scheme. Rabbits were bled at weeks 0, 3, 4, 8, 20, 36, 38, 41, 54, 56, and 57. Sera and PBMC samples were prepared and treated as described above for the protein immunization.

### 2.7. Antibody Assays

Sera were assessed for the levels of antibodies against HCV core-derived polypeptides and F-protein.

Core-derived peptides (Table 1) and core 147–191 were coated onto 96-well MaxiSorp plates and core polypeptides on the 96-well PolySorp plates (both from Nunc, Denmark). Coating was done overnight at 4°C in 50 mM carbonate buffer, pH 9.6, at antigen concentration of 10 *μ*g/mL. After blocking with PBS containing 1% BSA for 1 h at 37°C, serial dilutions of rabbit sera were applied on the plates and incubated for an additional hour at 37°C. Incubation was followed by three washings with PBS containing 0.05% Tween-20. Afterwards, plates were incubated for 1 h at 37°C with the protein A horseradish peroxidase-conjugated antibody (Sigma, USA) diluted 1 : 20000. Following three washes with PBS containing 0.05% Tween-20, the substrate OPD (Sigma, USA) was added, incubated at room temperature for 15 min in the dark, and stopped with 1 N H_2_SO_4_. Plates were read on an automatic reader (Multiscan, Sweden) at a dual length of 492 versus 630 nm. Immune serum was considered positive for anti-core antibodies whenever a specific OD value exceeded, by at least twofold, the signals generated by preimmune serum reacting with core-derived antigen and by immune serum reacting with BSA-coated wells.

### 2.8. PBMC Proliferation Assay

Peripheral mononuclear cells (PBMCs) were isolated by Ficoll Paque gradient centrifugation of blood which was collected in heparinized Vacutainer tubes. PBMCs were subjected to* in vitro* stimulation with core-derived synthetic peptides (Table 1) using the procedure described by us earlier [38]. In brief, T-cell proliferation assay was performed in triplicate with RPMI containing HCV core-derived peptides, all at 1 mcg/well; phytohemagglutinin (PHA; 10 mcg/well) was used as positive and RPMI alone and control peptide representing aa 605–613 of gp41 of HIV-1 were used as negative controls. Data were expressed as stimulation indices (SI) defined as the ratio of a mean value of [3H]-thymidine incorporation in the antigen-stimulated cultures to a mean value of radioactivity incorporation in medium containing negative control peptide from gp41 or RPMI, the highest of the values selected. SI values of 2.0 and above were considered positive. Data sets were discarded if SI by PHA was lower than 2.

### 2.9. Statistical Analysis

Statistical analysis was by paired Student's *t*-test, one-way ANOVA with pairwise comparisons, and two-way ANOVA with pairwise comparisons. *P* < 0.05 was considered significant. Analyses were performed using STATISTICA AXA 10.0.

## 3. Results and Discussion

### 3.1. Design and Expression of Proteins Encoded by the 5′ Terminus of HCV Genomic RNA

The full-length HCV core 1–191 is unstable and is quickly processed to a more stable shorter core aa 1–173 (core 1–173) [39]. We have chosen the latter as the immunogen and designed a recombinant core 1–173 of HCV 1b basing it on the isolate AD78P1 [30] with modifications that aimed to improve the prokaryotic expression (GenBank accession #KT824963). HCV core 1–173 is further degraded to the shorter forms, of which only core aa 1–152 (core 1–152) is readily detectable [40] motivating its choice as a second immunogen for the comparative immunogenicity studies. The expression of HCV core aa 1–152 variant was described by us earlier [32]. The panel of immunogens was complemented by the C-terminal fragment of HCV core aa 147–191 represented by a synthetic peptide (core 147–191).

The 5′ terminus of HCV RNA encodes also the proteins from the alternative reading frame (ARF). ARF of HCV lacks an in-frame AUG start codon; its expression involves unusual translation-level events involving ribosomal frameshifting [41]. ARF encoded proteins (ARFPs) are synthesized through multiple events and sites such as codons (in phase +1) 26, 42, 85/87, and 144 yielding different ARFP forms including double frameshifts [42–45]. Of those, the main most stable form is F-protein, whereas the rest are comparatively short and proteolytically unstable [46]. The frameshift leading to the production of ARFP/F is remarkable: it leads to the shutdown of the main ORF for at least one round of translation and occurs so frequently that it causes the ribosome to translate +1 reading frame approximately 30% of the time [47, 48]. This points at the abundance of F-protein and its significance as a target of HCV-specific immune response. We have chosen this longest and most stable ARFP form for the immunogenicity study in rabbits, to compare its immunogenic performance to that of the “classical” product of translation of the 5-terminus of HCV RNA. For this, we designed a recombinant protein containing the N-terminal 10 amino acids of HCV core and aa 11 to 143 belonging to F-protein of HCV 1b variant [31]. Only the first ten amino acids of HCV core were retained as they were shown to stabilize F-protein and support its correct folding [49]. One of the major antigenic sites of the core protein has been located away from the very N-terminus of HCV core (amino acids 9–16 [50]). Hence, we expected that sharing of the first ten amino acids will not interfere with the development of F-specific immune response.

**Figure 1:**
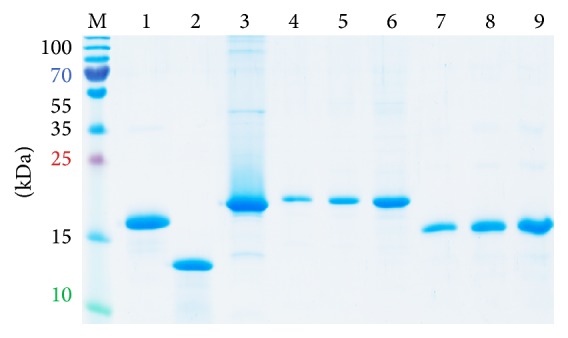
Expression of structural proteins encoded by the 5′ terminus of HCV RNA, HCV core aa 1–173 (lanes 4–6) and F-protein (lanes 7–9).* E. coli* were transformed with plasmids expressing core 1–173 and F-protein; cell lysates were resolved by 15% SDS-PAGE; gel was stained with Coomassie brilliant blue. HCV core 1–173 (0.5, 1, and 2.5 *µ*g per well, lanes 4–6) and F-protein (0.5, 1, and 2.5 *µ*g per well, lanes 7–9), respectively. Controls: His-tagged outer surface protein BB0689 of* B. burgdorferi* (2.5 *µ*g, lane 1), lysozyme (2.5 *µ*g, lane 2); HBcAg (2.5 *µ*g, lane 3); PageRuler Plus Prestained Protein Ladder (Thermo Scientific, lane M). Position of molecular mass markers is given on the left.

Core 1–173 and F-protein were expressed in* E. coli* with high yields (2–5 mg/L) and purified by His-tag chromatography. Coomassie staining of PAAG containing protein-rich fractions demonstrated the presence of proteins of expected molecular mass of 19 kDa for HCV core 1–173 (lanes 4–6) and of 16 kDa for F-protein (lanes 7–9) (Figure 1), in conformity with the observed products of translation of ARFs of HCV genotypes 1a, 1b, 1c, 2, and 3 [27, 51–55]. Proteins were of over 95% purity (Figure 1).

### 3.2. Polypeptides Derived from the 5′ Terminus of HCV RNA Induce Potent Antibody Response in Rabbits

**Figure 2:**
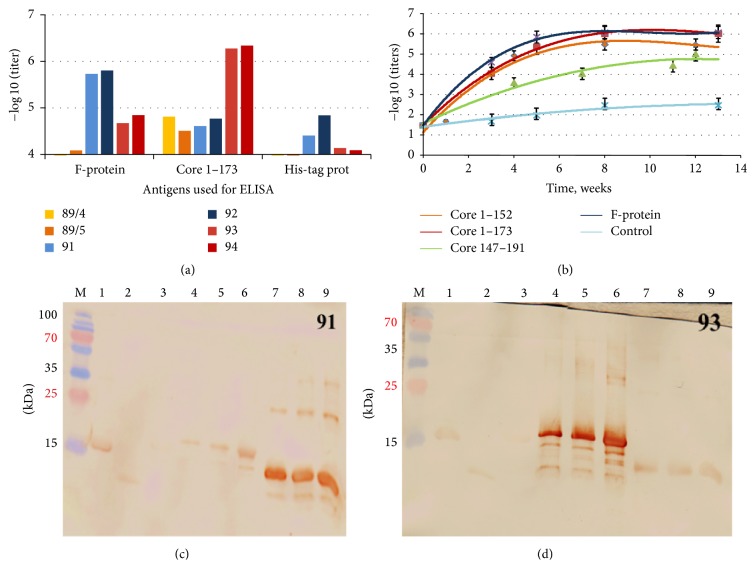
Antibody responses raised after immunization of rabbits with polypeptides encoded by the 5′ terminus of HCV genomic RNA. Maximum titer of antibodies against the immunogens (a); kinetics of the development of specific antibody response; controls represent rabbits mock-immunized with adjuvant alone; serum reactivity was tested by ELISA on plates coated with core 1–173 and F-protein (b); reactivity in Western blotting of hyperimmune sera raised against F-protein (serum of rabbit 91, (c)) and HCV core 1–173 (serum of rabbit 93, (d)). Western blotting was done with hyperimmune sera of rabbits collected by the end of immunization and diluted 1 : 10^4^. Lanes in panels (c and d) represent outer surface protein BB0689 of* B. burgdorferi* carrying 6xHis-tag (2.5 *µ*g, lane 1), lysozyme (2.5 *µ*g, lane 2), HBcAg (2.5 *µ*g, lane 3), core 1–173 (0.5, 1, and 2.5 *µ*g, lanes 4–6, resp.), and F-protein (0.5, 1, and 2.5 *µ*g, lanes 7–9, resp.). PageRuler Plus Prestained Protein Ladder (Thermo Scientific, lane M). Position of molecular mass markers is given on the left.

**Figure 3:**
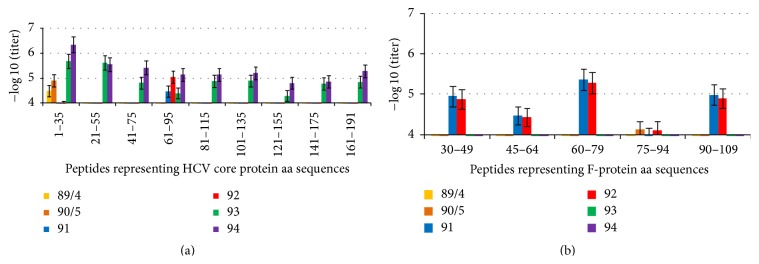
Fine epitope mapping of antibody response to linear epitopes of HCV core (a) and F-protein (b) recognized by rabbits immunized with HCV core aa 1–173 (nn 93, 94), HCV core 1–152 (89/4, 90/5), and F-protein (91, 92). Graphs demonstrate the highest antibody titers reached throughout immunization and represent the result of two to three independent ELISA runs. Unspecific antipeptide reactivity in control rabbits receiving adjuvant alone was below 5 × 10^2^.

Rabbits were immunized by the repeated injections of the polypeptides representing core aa 1–173 (core 173), core aa 1–152 (core 1–152), core aa 147–191 (core 147–191), and F-protein. All polypeptides were highly immunogenic on the humoral level; maximum antibody titers after completion of immunization cycle reached 10^6^ and the titer of antibodies to aa 147–191 reached over 10^5^ (Figure 2(a)). The strongest antibody response was achieved after immunization with HCV core 1–173 and F-protein (Figures 2(a) and 2(b)). HCV core 1–152 devoid of C-terminus generated a weaker antibody response with the maximum titer of 5 × 10^5^ despite an identical immunization scheme and almost identical antigen structure of the proteins (except for the lack of C-terminus) (Figures 2(a) and 2(b)). A 44-amino-acid long core 147–191, although used in immunization without carriers (which normally ensure strong antibody response against the synthetic peptides), induced a strong specific immune response with the titers reaching 10^5^ and the same kinetics of the antibody response as the longer polypeptides (Figures 3(a) and 3(b)). No anti-HCV core or anti-F-protein antibodies were detected in control rabbits 95, 96 receiving adjuvant alone (data not shown).

Sera raised against F-protein, core 1–152, and core 1–173 specifically recognized the respective recombinant proteins in Western blotting (Figures 2(c) and 2(d) and data not shown). Core 1–173 and F-protein specific sera demonstrated also a weak cross-reactivity (Figures 2(c) and 2(d) and Supplementary Figure S1 in Supplementary Material available online at http://dx.doi.org/10.1155/2015/762426). The latter can be attributed to the presence in both proteins of 6xHis-tag. Indeed, we showed rabbits to develop antibodies against anti-His-tag in titer of 10^4^ to 5 × 10^4^ (Figure 2(a)).

We have used a panel of synthetic peptides (Table 1) to map the B-cell epitopes of HCV core and F-protein recognized in rabbits. In HCV core aa 1–173, nine epitopes were identified which were distributed throughout the protein with the dominant region located at N-terminus of the protein (Figure 3(a)). The sera of core 1–152 immunized rabbits recognized only the immunodominant epitope at aa 1–35 (titer 5.5 × 10^4^, Figure 3(a)). Similar analysis was performed for the epitopes of F-protein (Figure 3(b)). B-cell epitopes of F-protein recognized in rabbits were localized at aa 30–49, 45–64, 60–79, and 90–109 (Figure 3(b)). The titer of antibodies against linear epitopes of F-protein was on the average 10-fold lower than against the linear epitopes of HCV core indirectly indicating a dominance of the HCV core-specific immune response over that against F-protein, at least in the rabbit model. The analysis of B-cell reactivity against HCV core and F-protein in rabbits uncovered similarity to the B-cell responses observed in HCV infection [27, 50, 54, 56–59]. Most of these epitopes were also shown to be recognized in mice [60, 61]. This reveals a promiscuous character of HCV core and F-specific B-cell response. Our findings also indicate that the recombinant F-protein obtained here is immunologically identical to the one formed after translation of viral RNA in infection and can be utilized in the diagnostic and possibly vaccine studies.

We have further characterized the nature of cross-reactivity between anti-HCV core and anti-F-protein sera seen in Western blotting (Figures 2(c) and 2(d)). The cross-reactivity of anti-HCV core 1–173 and anti-F-protein sera amounted to 10% of the total reactivity of both HCV core and F-protein immunized rabbits (Supplementary Figure S1). Immunization with HCV core 1–173 did not induce any antibodies reacting with F-protein-derived peptides. Immunization with F-protein did not induce an immune response reacting to core peptides except for the region aa 61–95 (Figure 3(a)). Analysis of the sequences of HCV core 1–173 and F-protein did not reveal any amino acid homologies, indicating that cross-reacting anti-F-protein antibodies might have recognized not a linear but a conformational epitope at aa 61–95 which could be reproduced by the synthetic peptide. Indeed, preblocking with the peptide encompassing aa 61–95 had no effect on the cross-reactivity of anti-F-protein sera with core 1–173 in Western blotting (i.e., anti-F-protein antibodies reacting to the peptide core aa 61–95 in ELISA were unable to recognize this sequence in the context of the denatured core 1–173; data not shown). Importantly, although 10 amino acids overlap between HCV core 1–173 and F-protein at N-terminus, anti-F-protein sera did not recognize synthetic peptide representing aa 1–35 of HCV core (Figure 3(b)). Altogether, this indicated that the cross-reactivity was apparently due to the immune recognition of His-tag.

Thus, all polypeptides derived from the 5′ terminus of HCV genomic RNA were found to be extremely immunogenic on the antibody level. Furthermore, we have demonstrated a similarly strong immunogenicity of the HCV core and F-proteins. Albeit no function has yet been attributed to F-protein (or other products of ARFPs), it represents a target of immune response equal in potency to HCV core [26, 54, 62]. Supposedly nonfunctional but abundant ARFPs may induce a decoy response leading to the immune system away from addressing “the meaningful” viral proteins; its high immunogenicity in rabbits confirms a possibility of their competition in the induction of antiviral immune response. This would fall in line with the recent model of Skums et al. which suggests antigenic cooperation in HCV infection, with immune responses against one antigen variant creating protective immune environment for other variants [63].

**Figure 4:**
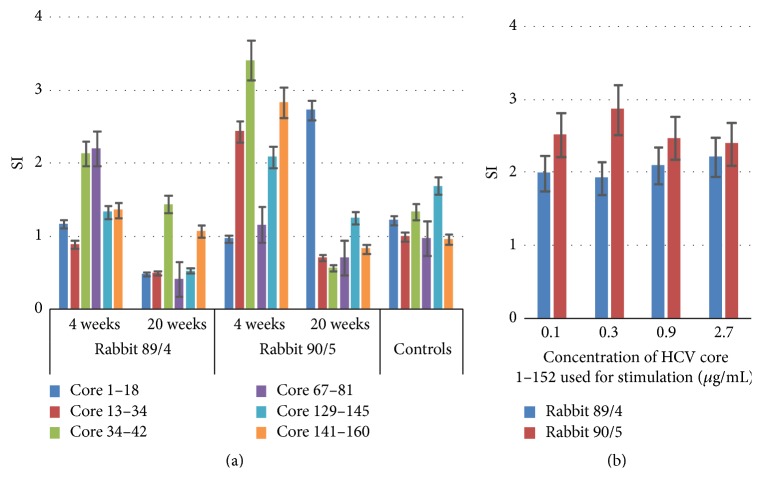
Proliferative response to HCV core in rabbits immunized with HCV core 1–152 visualized as stimulation indexes (SI). Stimulation of PBMCs of rabbits 89/4 and 90/5 with synthetic peptides derived from core 1–152 two weeks after prime (week 4) and two weeks after the last boost (week 20); controls are naïve rabbits (*n* = 3) and rabbits were immunized with irrelevant protein antigens (*n* = 4) (a); low or no dependence of T-cell stimulation on the concentration of HCV core 1–152 used in proliferation test in the test done prime (week 4) (b). All antigen stimulation tests were performed in triplicate. Test results were discarded if radioactivity incorporation values demonstrated by mitogen PHA were below 1000 counts per minute and if stimulation indexes in response to PHA were below 2.

Since F-protein has not yet been ascribed any function in the virus, while functions of HCV core are well known and essential, we concentrated our further immunogenicity studies on the cellular immune response against HCV core. Peripheral blood mononuclear cells (PBMCs) of rabbits immunized with HCV core 1–152 and core 1–173 were collected prior to and after each boost and subjected to stimulation with HCV core-derived peptides. Weak infrequent T-cell responses with stimulation indexes (SI) exceeding 2 were repeatedly observed only in rabbits immunized with HCV core 1–152, but not in the naïve or adjuvant or core 1–173-immunized animals (Figure 4, data not shown). Proliferative response of rabbit PBMCs was observed after two priming HCV core 1–152 immunizations (week 4) and was not boosted except for a single response to the epitope at the HCV core N-terminus observed in the rabbit 90/5 at week 20 (Figure 4(a)). Stimulation of the hyperimmune rabbit PBMCs with recombinant HCV core 1–152 induced a weak proliferative response independent of antigen concentration (Figure 4(b)).

Analysis of HCV core-specific humoral and cellular responses revealed that the C-terminally truncated HCV core form had somewhat weaker humoral immunogenicity than HCV core aa 1–173: antibodies were two to three times lower in titer and of restricted specificity targeting mainly the N-terminus of the protein (Figures 2 and 3). At the same time, only core 1–152 was able to induce a specific T-cell response, albeit of a very low level. Apparently, the truncation of the C-terminus led to a partial loss of B-cell immunogenicity (in terms of both breadth and potency) and at the same time the induction of the T-cell arm of immune response. We have recently shown that HCV core devoid of the N-terminus upregulated the transcription of a ROS-generating enzyme cytochrome P450 2E1 [64]. Furthermore, the same fragment induced the expression of endoplasmic reticulum oxidoreductin 1*α*. The latter triggers the efflux of Ca^2+^ ions from ER to mitochondria via mitochondrial Ca^2+^ uniporter, leading to the generation of superoxide anions and possibly also H_2_O_2_ [64]. ROS have a physiological role in signaling extending to every cell type involved in the induction of immune response; ROS were the first molecules found to suppress the T-cell function [65]. As with any signaling mechanism, ROS can become cytotoxic if the signal is too strong and/or too prolonged. ROS help to mediate T-cell activation; however, T-cell activation also depends on the capacity of accessory cells to maintain sufficient level of glutathione and is compromised by the oxidative stress [66]. Furthermore, excessive amounts of ROS can oxidize the protein kinases and phosphatases that regulate critical cell signals and distort the activation of signaling pathways including regulation of the lymphocyte functions [67]. Immunosuppressive effects of ROS may also be due to the fact that Tregs cells are more resistant to ROS than the effector T-cells and pertain their downregulating activity when the effector T-cells fail [66]. An excessive oxidative stress may thus be detrimental for the normal T-cell functions. This would explain the observed deleterious role of the high HCV core protein “doses” for the specific T-cell immunity in a mouse model [9]. For humoral response, on the contrary, ROS appear to contribute to Th1/Th2/Th17 cell fate decisions during T-lymphocyte activation and enhance immunoglobulin production by B-lymphocytes [68]. Our data indicates that (as in HCV infection) high levels of HCV core support the strong multiepitopic B-cell, but low or no T-cell response, and point at the role of certain core domains, specifically at the C-terminus, in tilting the response towards the humoral one. Involvement in ROS induction may explain an unexpectedly strong humoral immunogenicity in rabbits of a peptide covering aa 147–191 derived from the ROS-inducing core fragment.

### 3.3. Immunogenicity in Rabbits of DNA Encoding Core aa 1–152

High levels of circulating HCV core antigen in HCV infection would induce high levels of ROS and promote strong humoral response but little immunity on the T-cell level, a scenario of immune response in the chronic HCV infection, whereas the immune success and viral clearance coincide with a weak or no antibody response [69] and potent cellular immunity manifested mainly CD4+ T-cells [13, 17]. To strengthen the cellular immune response component, one would need to both decrease the immunogen dose and delete the ROS-inducing/B-cell activating signals.

**Figure 5:**
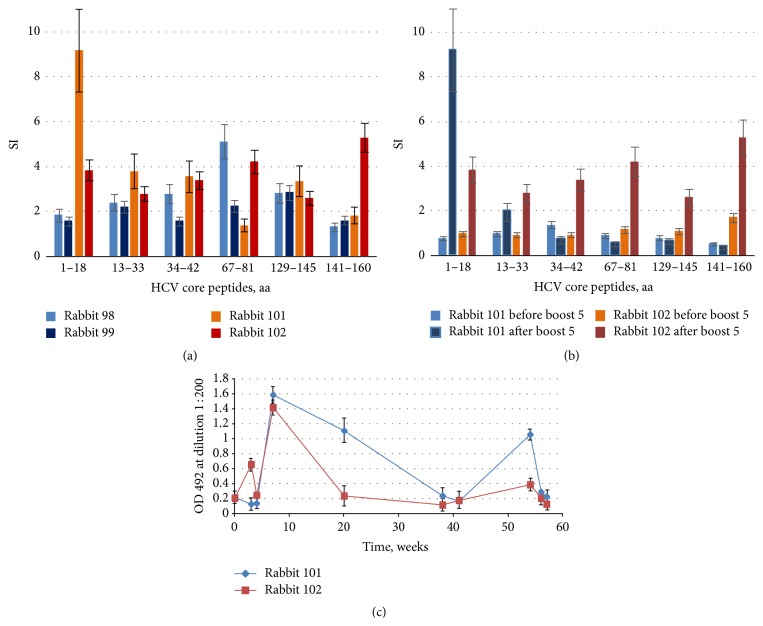
Anti-HCV core immune response induced by single and repeated immunizations with synthetic gene encoding core aa 1–152 (DNAcore152). Rabbits were regularly bled; PBMCs were isolated and subjected to stimulation with HCV core-derived peptides and recombinant HCV core aa 1–152. Stimulation indexes (SI) observed after double priming (week 4) (a); boosting of T-cell response in rabbits receiving multiple injections of DNAcore152 illustrated by stimulation indexes exhibited by PBMCs of rabbits 101 and 102 before and after boost 5 at weeks 54 and 56, respectively (b); dynamics of antibody response to HCV core aa 1–152 in rabbits receiving repeated injections of DNAcore152 (c). All antigen stimulation tests were performed in triplicate; SI values represent an average with standard deviation. Test results were discarded if radioactivity incorporation values demonstrated by mitogen PHA were below 1000 counts per minute and if stimulation indexes in response to PHA were below 2. HCV core-specific antibodies response represent an average optical density exhibited by sera of each of the rabbits collected at given time points in two ELISA runs with standard deviations. OD of sera of rabbits immunized with empty vector DNA collected at the same time points did not exceed the optical density of 0.3 (data not shown).

To evade both the potential pitfalls as immune suppression induced by an excess of ROS and the immune competition from the ARF products, we exploited a synthetic gene encoding HCV core devoid of the C-terminus (DNAcore152) with a forbidden frameshift (not supporting F-protein formation [36]). Rabbits were immunized with DNAcore152 by two closely spaced priming injections (double prime, four rabbits), in two rabbits followed by a series of boosts performed first with one-month and then with four-month intervals. The latter scheme was applied in view of earlier experiments in chimpanzees which demonstrated gradual increase if there were proliferative responses to HCV core after repeated boosts performed under long period of time [4]. Contrary to the HCV core 1–152 immunized rabbits, rabbits receiving injections of DNAcore152 exhibited low but consistent proliferative response to both HCV core and core-derived peptides (Figure 5(a)), boosted by the booster DNA injections (Figure 5(b); Supplementary Figure S2). Rabbits immunized with repeated injections of DNAcore152 developed also a low-level humoral response to HCV core, weakly boosted after the repeated gene administrations (Figure 5(c)). No anticore responses were registered in rabbits mock-immunized with empty vector DNA (data not shown).

Immunization with DNA encoding HCV core devoid of the 39 amino acids on the C-terminus allowed shifting the immune response to almost exclusively Th1 type as manifested by weak but consistent core-specific proliferative responses of PBMCs and low level of anti-core antibody production resembling the profiles observed in the primate trials of the multicomponent immunogens including diverse forms of HCV core (DNA, recombinant virus, protein) [4–6]. These results fall in line with our earlier observations made in the DNA-immunized mice; namely, the induction of cellular response to HCV core does not require high levels of HCV core protein (low amounts provided by cells* in vivo* transfected at immunization sites appear to be sufficient). Furthermore, we could show that truncation, at least partial, of the ROS-inducing core domain may rescue cellular response. Additional positive input was possibly made by forbidding the formation of F-protein. The actual role of immune competition from the F-protein is currently being assessed in a series of mouse immunizations.

Again, as in the case of protein immunizations, the specificity of B- and T-cell response to HCV core-derived peptides in DNA-immunized rabbits resembled that observed in the HCV infection [70]. Core-specific T-cell responses were persistent and boostable, resembling responses observed in the self-limiting rather than chronic HCV infection which is characterized by a gradual loss of the specific T-cell response [17, 71].

## 4. Conclusions

In primate trials, the responses to the structural HCV proteins including the nucleocapsid (core) were shown to significantly correlate to the protection against HCV challenge [1] implying an input of anticore response on the observed protection effects. This emphasizes the necessity of the experiments aimed at achieving an effective core-specific cellular response in larger animals than mice. Rabbits are widely used in the toxicity and safety testing of medical devices, drugs, and vaccines because of both genetic heterogeneity and possibility of the longitudinal follow-up experiments. Here, we used the rabbit model to evaluate the immunogenicity of polypeptides encoded by the 5′ terminus of HCV RNA. By polypeptide immunization, we have induced in rabbits a strong humoral immune response to an abundant HCV core form aa 1–173 and the most stable ARFP form, F-protein. Immunization with HCV core aa 1–173 led to a B-cell response of broad specificity targeting multiple linear epitopes. The C-terminally truncated core 1–152 induced a weaker antibody response directed only against the N-terminus of the protein implicating the role of the C-terminus in promoting humoral immunogenicity. Delivery of the C-terminally truncated HCV core by DNA immunization with a plasmid forbidding frameshift led to the induction of weak but sustained T-cell response to multiple epitopes within the protein. Both B- and T-cell responses observed in rabbits mimicked that in HCV infection which indicates the promiscuity of major epitopes localized in the polyproteins encoded by the 5′-terminus of HCV genomic RNA. These are promising findings which allow a step forward in the development of the HCV core based prototype HCV vaccines, as the previous data indicated that although HCV core is the main target of an immune response in the infected individuals [72], it is not so immunogenic in the larger species as humans [73] and could even suppress the immune response [14], also heterologous [74]. The immunogenicity of DNA representing the 5′ terminus of HCV RNA and of the polyproteins encoded therein and the promiscuity of the observed responses promote the use of rabbit model for the preclinical trials of HCV vaccines, although other adjuvants would be needed to comply with the requests to vaccine formulations. These considerations may be important in further development and testing of HCV vaccines based on the structural viral proteins.

## Supplementary Material

Supplementary Figure S1: Analysis of reactivity in Western blotting of hyperimmune sera raised against the F-protein (rabbit 91), and HCV core1-173 (rabbit 93) using ImageJ software (). HCVcore1-173 and F-proteins in amounts of 1 to 2,5 ug were resolved by PAGE, transferred on nitrocellulose membrane, and membranes were stained by the sera of rabbits immunized with HCVcore1-173 (rabbit 93) or F-protein (91). Dose dependence of staining by hyperimmune serum of rabbit 93 of the 19kDa protein band corresponding to HCV core1-173 (lanes 4-6, Fig 2D) (A); Cross-reactivity of the end-point serum of the F-protein immunized rabbit N°91 with core1-173, and of the core1-173 immunized N°93 with core1-173 evaluated in the immune staining of 1 ㎍ of the antigens, presented as % of the total immune staining (corresponds to lanes 5 and 8 on Figures 2 C and D) (B).
Supplementary Figure S2: Dynamics of T-cell responses to synthetic peptides representing HCV core in rabbits 98 (A) and 99 (B) receiving DNAcore152 as double primes on weeks 0 and 1, and rabbits 101 (C) and 102 (D) receiving DNAcore152 as double primes followed by boosts on weeks 5, 18, 37 and 54. All antigen stimulation tests were performed in triplicates. Data represent an average stimulation index (SI) of rabbit PBMC demonstrated in each of the tests. Test results were discarded if radioactivity incorporation values demonstrated by mitogen PHA were below 1000 counts per minute, and if stimulation indexes in response to PHA were below 2.

## Figures and Tables

**Table 1:** A panel of overlapping peptides derived from HCV core and F-protein used in the tests of humoral and cellular immune response. First and last amino acid position are given according to HCV AD78 isolate genotype 1b (GenBank accession number AJ132996 [30, 36]).

Protein	Amino acid positions	Amino acid sequence
HCV core	1–35	MSTNPKPQRKTKRNTNRRPQDVKFPGGGQIVGGVY
	21–55	DVKFPGGGQIVGGGVYLLPRRGPRLGVRATRKTSER
	41–75	GPRLGVRATRKTSERSQPRGRRQPIPKARRPEGRT
	61–95	RRQPIPKARRPEGRTWAQPGYPWPLYGNEGMGWAG
	81–115	YPWPLYGNEGMGWAGWLLSPRGSRPSWGPNDPRRR
	101–135	RGSRPSWGPNDPRRRSRNLGKVIDTLTCGFADLMG
	121–155	KVIDTLTCGFADLMGYIPLVGAPLGGAARALAHGV
	161–195	GVNYATGNLPGCSFSILLALLSCLTTIPASAYEVR
	1–18	MSTIPKPQRKTKRNTNRR
	13–33	RNTNRRPQDVKFPGGGQIVGG
	34–42	VYLLPRRGP
	67–81	KARRPEGRTWAQPGY
	129–145	GFADLMGYIPLVGAPLG
	141–160	GAPLGGAARALAHGVRVLED

F-protein	30–49	SLAEFTCCRAGAPGWACARL
	45–64	ACARLGRLPSGRNLVEGDNL
	60–79	EGDNLSPRLAIPRAGPGLSL
	75–94	PGLSLGTLGPSMAMRAWGGQ
	90–109	AWGGQDGSCHPVALGLVGAP
