# Supplementary material for: Comparative Immunogenicity in Rabbits of the Polypeptides Encoded by the 5′ Terminus of Hepatitis C Virus RNA
Source: J Immunol Res. 2015 Nov 2;2015:762426. doi: 10.1155/2015/762426 (PMC4644844; doi:10.1155/2015/762426)
Supplement: Supplementary file 1 — Supplementary Figure S1: Analysis of reactivity in Western blotting of hyperimmune sera raised against the F-protein (rabbit 91), and HCV core1-173 (rabbit 93) using ImageJ software (). HCVcore1-173 and F-proteins in amounts of 1 to 2,5 ug were resolved by PAGE, transferred on nitrocellulose membrane, and membranes were stained by the sera of rabbits immunized with HCVcore1-173 (rabbit 93) or F-protein (91). Dose dependence of staining by hyperimmune serum of rabbit 93 of the 19kDa protein band corresponding to HCV core1-173 (lanes 4-6, Fig 2D) (A); Cross-reactivity of the end-point serum of the F-protein immunized rabbit N°91 with core1-173, and of the core1-173 immunized N°93 with core1-173 evaluated in the immune staining of 1 ㎍ of the antigens, presented as % of the total immune staining (corresponds to lanes 5 and 8 on Figures 2 C and D) (B). Supplementary Figure S2: Dynamics of T-cell responses to synthetic peptides representing HCV core in rabbits 98 (A) and 99 (B) receiving DNAcore152 as double primes on weeks 0 and 1, and rabbits 101 (C) and 102 (D) receiving DNAcore152 as double primes followed by boosts on weeks 5, 18, 37 and 54. All antigen stimulation tests were performed in triplicates. Data represent an average stimulation index (SI) of rabbit PBMC demonstrated in each of the tests. Test results were discarded if radioactivity incorporation values demonstrated by mitogen PHA were below 1000 counts per minute, and if stimulation indexes in response to PHA were below 2. [file 762426.f1.pdf]

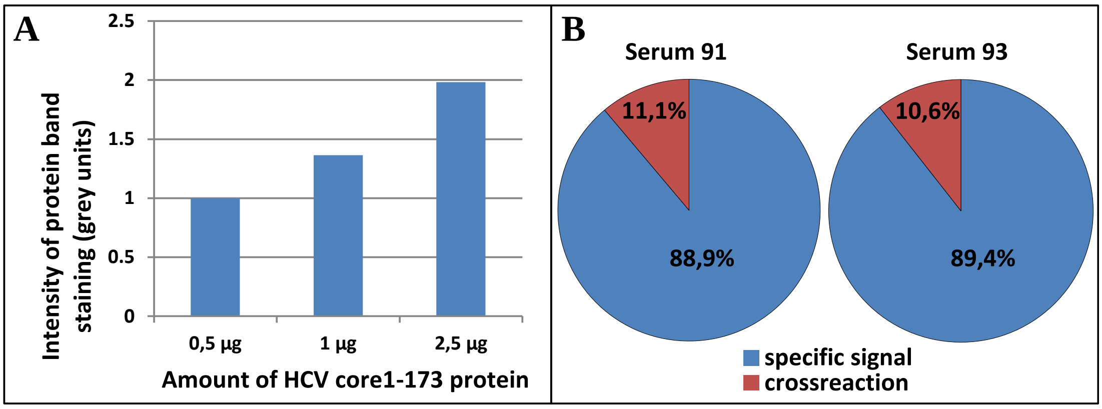

Supplementary Fig S1. Analysis of reactivity in Western blotting of hyperimmune sera raised against the F-protein (rabbit 91), and HCV core1-173 (rabbit 93) using *ImageJ* software (http://imagej.nih.gov/ij/). HCVcore1-173 and F-proteins in amounts of 1 to 2,5 ug were resolved by PAGE, transferred on nitrocellulose membrane, and membranes were stained by the sera of rabbits immunized with HCVcore1-173 (rabbit 93) or F-protein (91). Dose dependence of staining by hyperimmune serum of rabbit 93 of the 19kDa protein band corresponding to HCV core1-173 (lanes 4-6, Fig 2D) (A); Cross-reactivity of the end-point serum of the F-protein immunized rabbit №91 with core1-173, and of the core1-173 immunized №93 with core1-173 evaluated in the immune staining of 1 µg of the antigens, presented as % of the total immune staining (corresponds to lanes 5 and 8 on Figures 2 C and D) (B).

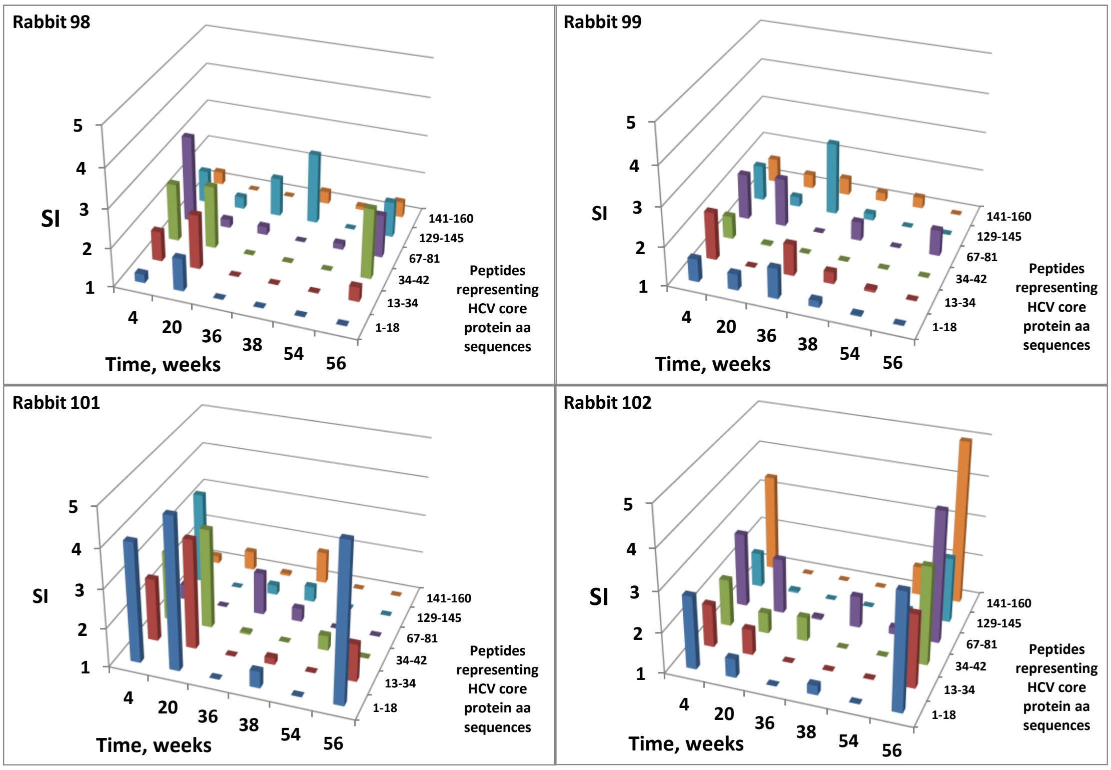

Supplementary Fig S2. Dynamics of T-cell responses to synthetic peptides representing HCV core in rabbits 98 (A) and 99 (B) receiving DNAcore152 as double primes on weeks 0 and 1, and rabbits 101 (C) and 102 (D) receiving DNAcore152 as double primes followed by boosts on weeks 5, 18, 37 and 54. All antigen stimulation tests were performed in triplicates. Data represent an average stimulation index (SI) of rabbit PBMC demonstrated in each of the tests. Test results were discarded if radioactivity incorporation values demonstrated by mitogen PHA were below 1000 counts per minute, and if stimulation indexes in response to PHA were below 2.
